# Interleukin-33 modulates inflammation in endometriosis

**DOI:** 10.1038/s41598-017-18224-x

**Published:** 2017-12-20

**Authors:** Jessica E. Miller, Stephany P. Monsanto, Soo Hyun Ahn, Kasra Khalaj, Asgerally T. Fazleabas, Steven L. Young, Bruce A. Lessey, Madhuri Koti, Chandrakant Tayade

**Affiliations:** 1 Department of Biomedical and Molecular Sciences, Queen’s University, Kingston, Ontario K7L 3N6 Canada; 2 Department of Obstetrics, Gynecology and Reproductive Biology, Michigan State University College of Human Medicine, Grand Rapids, MI 49503 USA; 3 Department of Obstetrics and Gynecology, University of North Carolina, Chapel Hill, North Carolina, NC 27514 USA; 4 Department of Obstetrics and Gynecology, Greenville Health Systems, Greenville, South Carolina, SC 29605 USA

## Abstract

Endometriosis is a debilitating condition that is categorized by the abnormal growth of endometrial tissue outside the uterus. Although the pathogenesis of this disease remains unknown, it is well established that endometriosis patients exhibit immune dysfunction. Interleukin (IL)-33 is a danger signal that is a critical regulator of chronic inflammation. Although plasma and peritoneal fluid levels of IL-33 have been associated with deep infiltrating endometriosis, its contribution to the disease pathophysiology is unknown. We investigated the role of IL-33 in the pathology of endometriosis using patient samples, cell lines and a syngeneic mouse model. We found that endometriotic lesions produce significantly higher levels of IL-33 compared to the endometrium of healthy, fertile controls. *In vitro* stimulation of endometrial epithelial, endothelial and endometriotic epithelial cells with IL-33 led to the production of pro-inflammatory and angiogenic cytokines. In a syngeneic mouse model of endometriosis, IL-33 injections caused systemic inflammation, which manifested as an increase in plasma pro-inflammatory cytokines compared to control mice. Furthermore, endometriotic lesions from IL-33 treated mice were highly vascularized and exhibited increased proliferation. Collectively, we provide convincing evidence that IL-33 perpetuates inflammation, angiogenesis and lesion proliferation, which are critical events in the lesion survival and progression of endometriosis.

## Introduction

Endometriosis is a chronic inflammatory, estrogen-dependent disease that affects 6–10% of reproductive-aged women. Despite this considerable prevalence, the cause of endometriosis remains unknown and a cure does not exist. Retrograde menstruation is a widely accepted theory to explain the pathogenesis. This theory suggests that during menstruation, uterine contractions cause menstrual endometrial tissue to be refluxed into the oviducts and peritoneal cavity. It was shown that 76–90% of all women experience this reflux of menstrual debris^1^. However, only in endometriosis patients is this menstrual tissue able to adhere to peritoneal structures, develop a blood supply and grow into an endometriotic lesion. Therefore, it is likely that the women who are developing endometriosis have genetic, biochemical or immune system dysfunction that does not allow the removal of the debris but rather facilitates menstrual tissue adhesion to peritoneal structures and endometriotic lesion formation^2,3^. Indeed, it is well established that women with endometriosis exhibit immune dysfunction in the form of heightened local and systemic inflammation^2,4,5^. More specifically, in the plasma and peritoneal fluid (PF), endometriosis patients display aberrant numbers of immune cells and concentrations of cytokines and chemokines that promote a chronic inflammatory environment compared to healthy women^6–10^. The chronic inflammatory environment has also been shown to contribute to the chronic pain and infertility experienced by endometriosis patients^3,11^.

Interleukin(IL)-33 is an alarmin of the IL-1 family that acts upon both the innate and adaptive immune system and plays functional roles in both infectious and chronic inflammatory diseases^12–15^. IL-33 is constitutively expressed in the nucleus of various cell types including endothelial cells and epithelial cells^16^; however, upon tissue damage, necrosis or mechanical stress, functional IL-33 is released into the extracellular environment and specifically binds to the ST2 receptor (suppressor of tumorigenicity 2)^17,18^. This receptor has two forms: a membrane bound form that initiates signaling (ST2) and a soluble, decoy receptor (sST2). IL-33 has been shown to stimulate both myeloid and lymphoid immune cells through ST2 including but not limited to macrophages, mast cells, T cells, B cells, NK cells, neutrophils, and innate lymphoid cells^12,19–22^. Binding of IL-33 to ST2 induces signaling by recruiting MyD88 and transcription factors such as NF-KB through IRAK1/4 and TRAF6 kinases to perpetuate inflammation: primarily a type 2 immune response^23^. Endometrial stromal cells produce IL-33 specifically during decidualization suggesting an important role in female reproductive events such as embryo implantation and early pregnancy^24^.

In the literature surrounding endometriosis and IL-33, there are only two reports so far. In a study conducted in 532 endometriosis patients and 130 women without endometriosis, IL-33, in serum and PF samples, was significantly higher in patients with a deep infiltrating endometriosis phenotype compared to control women^25^. In another study conducted in 30 endometriosis patients and 20 control women from Tunisia, IL-33 was reported to be higher in the serum and PF of women with endometriosis^26^. Furthermore, sST2 concentrations were significantly higher in PF from endometriosis patients compared to controls^26^. Additionally, they show that IL-33 mRNA expression was higher in cells obtained from PF from endometriosis patients compared to healthy controls^26^. These reports suggest that women endometriosis, specifically with deep infiltrating endometriosis, have high levels of IL-33 in their PF and plasma but they do not suggest whether IL-33 is playing an active role in the disease progression or if it is a bystander. Interestingly, IL-33 is a critical regulator of number of processes including inflammation, vascularization, hypernociception, and fibrosis^27–30^, which are known to be involved in the pathophysiology of endometriosis. Therefore, it is possible that IL-33 plays a role in the progression of endometriosis; however, a significant knowledge gap exists with regards to how IL-33 contributes to the disease pathology and which tissues and cells are producing IL-33. In the present study, we show for the first time that endometriotic lesions contribute to the production of IL-33 and that levels of IL-33 are significantly higher in the endometriotic lesions of advanced staged patients compared to the endometrium of healthy, fertile controls. We also show that endometriotic lesions express the ST2 receptor. Using a series of *in vitro* studies in cell lines, we documented the effects of human recombinant IL-33 (rIL-33) on the proliferation, angiogenesis and cytokine profile. Finally, we show that mouse rIL-33 induces local and systemic inflammation, increases the lesion size and increases vascularization within the lesion itself in a syngeneic endometriotic mouse model.

## Results

### Endometriotic (ectopic) lesions produce IL33 and express the ST2 receptor

**Figure 1:**
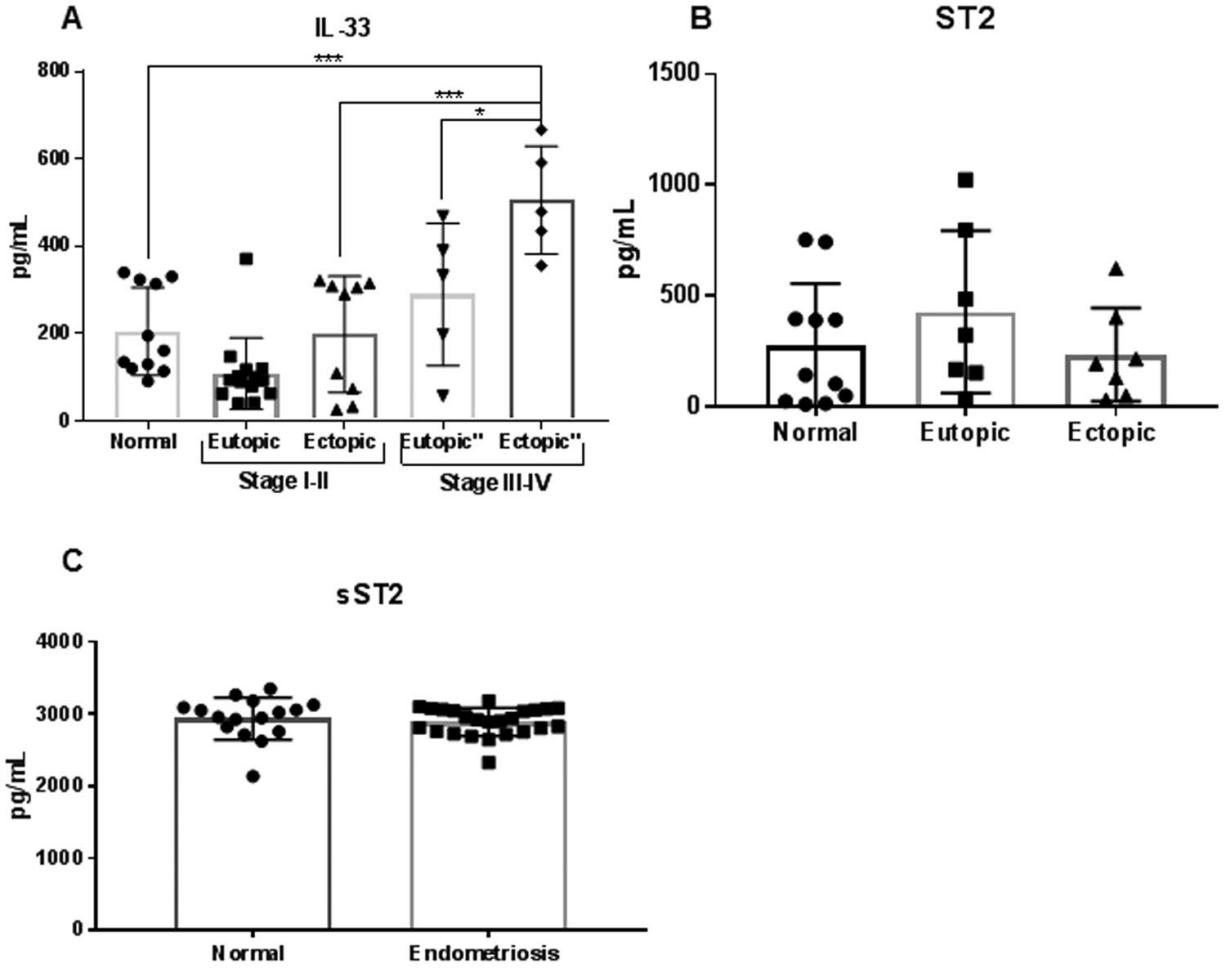
Protein levels of IL-33, ST2 and sST2 in plasma and tissue samples from healthy, fertile volunteers and endometriosis patients. (**A**) Levels of IL-33 are significantly higher in the ectopic tissue from advanced staged endometriosis patients (n = 5) compared to healthy fertile controls (n = 11). No significant differences were found between healthy, fertile controls compared to early staged patients (n = 14) or eutopic tissue from advanced staged patients (n = 5). (**B**) The ST2 receptor is expressed on ectopic, endometriotic tissue. No significant differences were found between levels of ST2 in matched tissue from endometriosis patients (n = 7) compared to healthy, fertile controls (n = 11). (**C**) No significant differences were found between levels of sST2 in the plasma of endometriosis patients (n = 24) compared to healthy, fertile controls (n = 16) *p < 0.05 **p < 0.01 ***p < 0.0001.

Previous reports indicated high levels of IL-33 in the serum and PF of deep infiltrating endometriosis patients, the source of IL-33 was not clear. We show for the first time that the endometriotic lesions from stages III and IV patients produce significantly higher levels of IL-33 compared to endometrial tissue from healthy, fertile controls and that the endometriotic lesions from these advanced staged patients produce significantly more IL-33 than matched tissue from their endometrium (Fig. 1A). We also report that endometriotic lesions express the ST2 receptor (Fig. 1B). No significant differences were found in the expression of ST2 between endometriosis patient tissue and tissue from healthy, fertile controls (Fig. 1B). Additionally, we did not find any significant difference in the levels of sST2 in the plasma of endometriosis patients compared to healthy fertile controls (Fig. 1C).

### Human rIL-33 stimulates angiogenic and proinflammatory cytokines production

**Figure 2:**
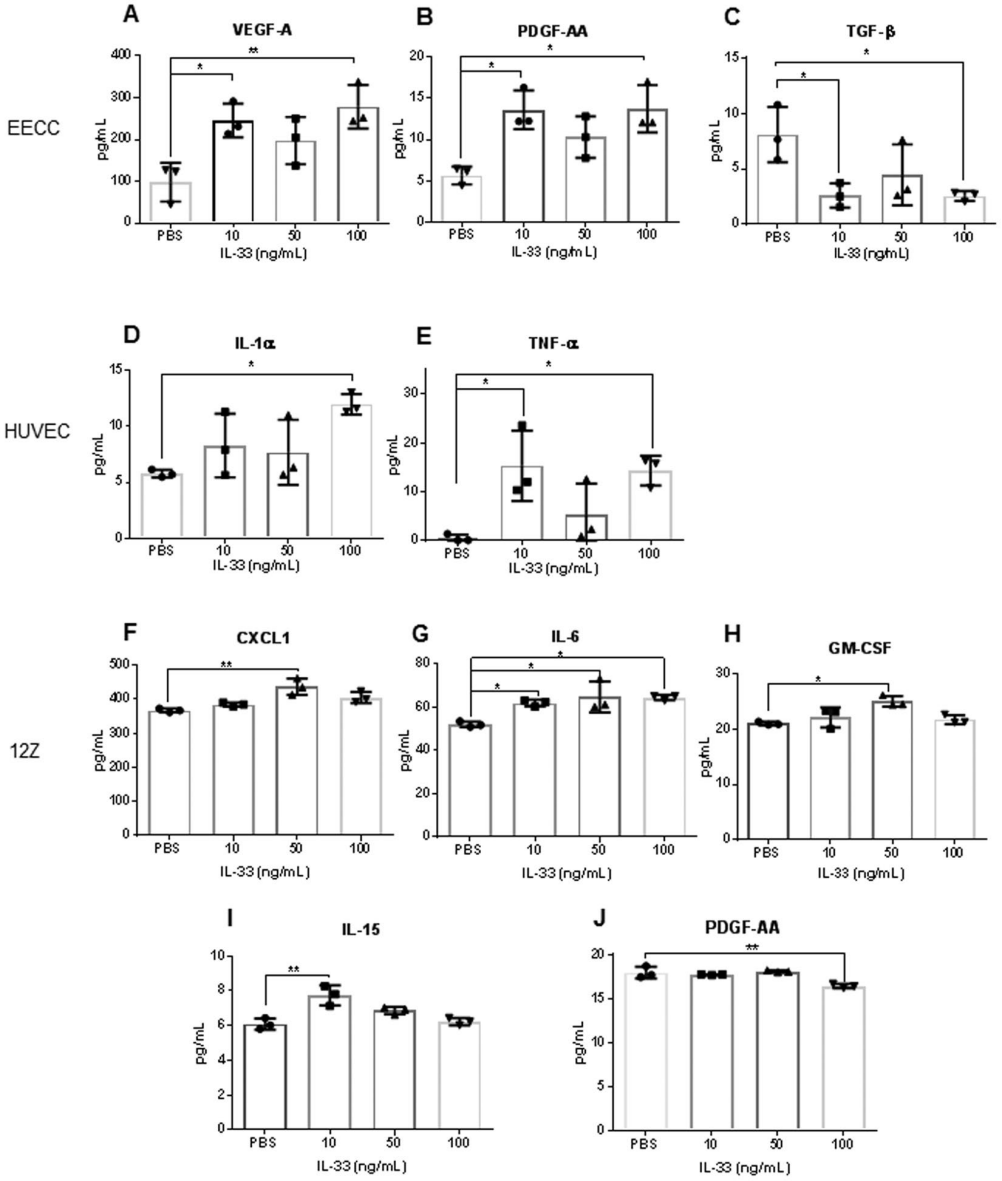
Cell supernatant cytokine profile in EECC, HUVEC and 12Z upon stimulation with varying concentrations of human rIL-33 (10, 50 and 100 ng/mL). All experiments were conducted in triplicates. Cell supernatant was analyzed using a human multiplex assay and non-significant cytokines are not shown. (**A**–**C**) Endometrial epithelial carcinoma cells (EECC) produced significantly higher levels of VEGF and PDGF-AA and a significant reduction in the level of TGF-b. (**C**,**D**) Human umbilical vein endothelial cells (HUVEC) produced significantly higher levels of IL-1a and TNF-a. (**F**–**J**) Endometriotic epithelial cells (12Z) produced significantly higher levels of CXCL1, IL-15, GM-CSF and IL-6 and significantly lower levels of PDGF-AA. *p < 0.05 **p < 0.01 ***p < 0.0001.

First, we confirmed using RT-PCR and sequencing that all three cell lines used in the study (EECC, HUVECs and 12Zs) express the ST2 receptor. Then, we sought to understand the effect of IL-33 signalling on the proliferation and cytokine production in these cell lines. Using IncuCyte, live cell analysis platform, we found that treating EECCs, HUVECs and 12Zs with varying concentrations of human rIL-33 (1 ng/mL, 10 ng/mL, 50 ng/mL and 100 ng/mL) did not have any effect on their proliferation (Supplemental Fig. 1). To assess the cytokine production of each cell line in response to human rIL-33, we screened the cell supernatants from EECCs, HUVECs and 12Zs treated with varying concentrations of rIL-33. Supernatants from EEECs revealed significantly higher (P < 0.005) levels of angiogenic cytokines including VEGF and PDGF-AA and significantly lower expression of TGF-β compared to PBS-treated cells (Fig. 2A–C). Treating HUVECs with human rIL-33 led to significantly higher (P < 0.05) concentration of IL-1α and TNF-α compared to PBS-treated cells (Fig. 2D,E). Treatment of the endometriotic epithelial cell line, 12Z, with human rIL-33 stimulated significantly higher (P < 0.05) production of pro-inflammatory cytokines such as CXCL1, IL-6, GM-CSF, and IL-15 compared to PBS treated cells (Fig. 2F–J). These results further confirm that IL-33 is a potent pro-inflammatory and angiogenic cytokine.

### Human rIL-33 stimulates tubulogenesis in Human Umbilical Venule Endothelial Cells

To confirm previous reports showing the angiogenic properties of IL-33^31^, we conducted a tubulogenesis assay using HUVECs in matrigel. HUVECs were stimulated with varying concentrations of human rIL-33 (10 ng/mL, 50 ng/mL, and 100 ng/mL) and total tubule branch length was measured. PBS was used as a negative control and VEGF was used as a positive control (Supplemental Fig. 2F).

### Recombinant IL-33 treatment in mouse model of endometriosis perpetuates systemic inflammation

**Figure 3:**
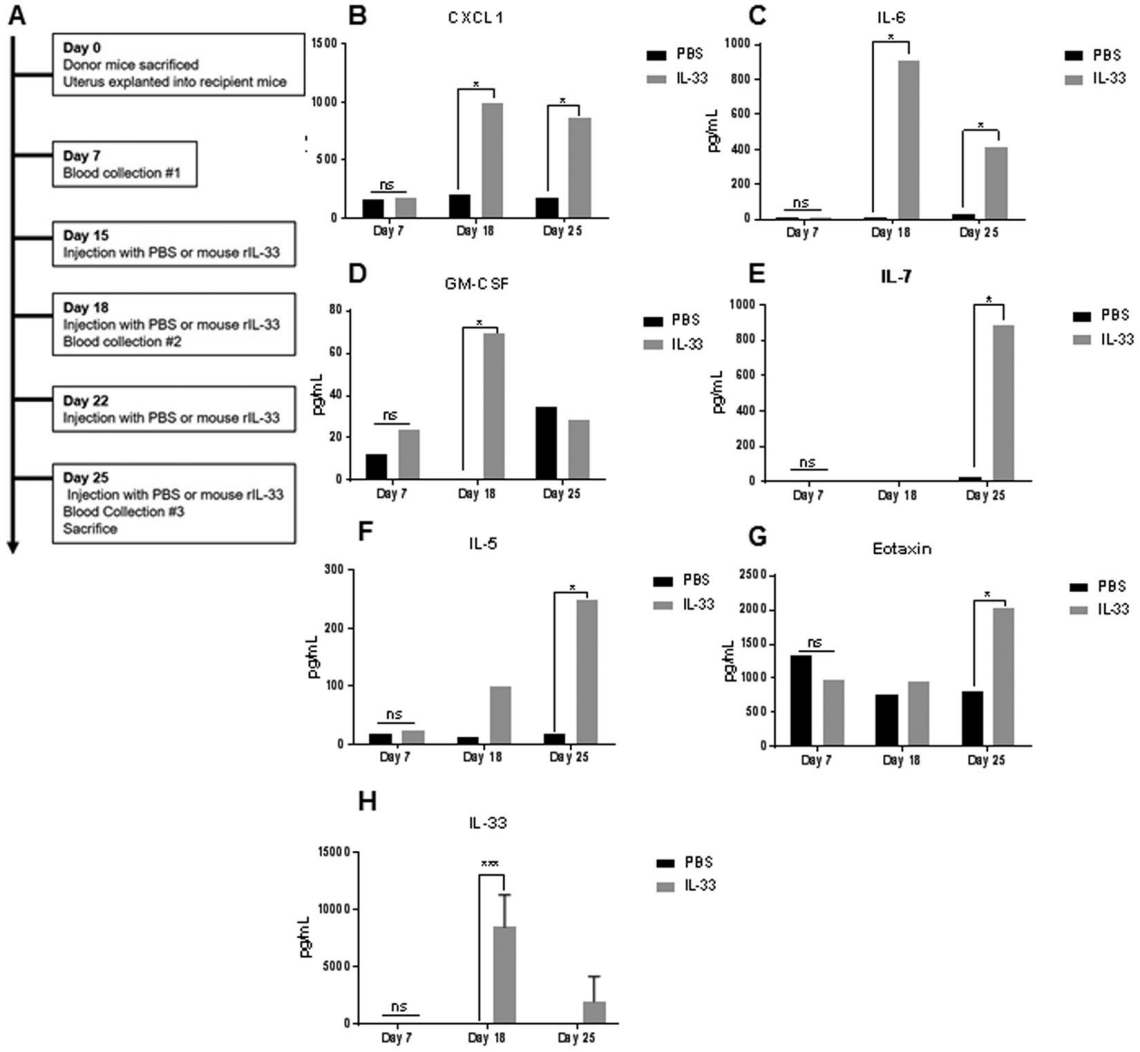
Plasma cytokine profile in mice with endometriosis and treated with PBS (control) (n = 5) or mouse rIL-33 (treated) (n = 5). Plasma was analyzed using a mouse multiplex assay and non-significant cytokines are not shown. (**A**) An outline of mouse experiment. (**B**–**G**) Plasma cytokines revealed significantly higher levels of Eotaxin, GM-CSF, IL-6, IL-7, IL-5, CXCL1, and IL-33. *p < 0.05 **p < 0.01 ***p < 0.0001.

To understand whether the elevated levels of IL-33 in the PF of deep infiltrating endometriosis patients^25^ contributes to the disease, we recreated heightened IL-33 environment in a syngeneic mouse model by injecting mouse rIL-33 into the peritoneal cavity. Because inflammation has been speculated to contribute to both the pathophysiology and symptoms of the disease, we used the plasma cytokine levels as an indicator to understand the effect of mouse rIL-33 on systemic inflammation. The plasma cytokine levels were analyzed at three different time points (day 7, 18 and 25) using a mouse cytokine multiplex assay (Fig. 3A). On day 7, before the start of injections, both treatment and control mice showed similar levels of plasma cytokines. Following injections of mouse rIL-33, we observed significantly higher levels of CXCL1, IL-6, GM-CSF, Eotaxin, IL-5, IL-7 and IL-33 compared to control mice treated with PBS (Fig. 3B–F).

We also evaluated whether rIL-33 would modulate inflammation in mice in the absence of endometriotic lesions. The plasma samples obtained from C57Bl6 mice treated with rIL-33 had elevated levels of CXCL1, IL-6, GM-CSF, IL-7, IL-5 and Eotaxin (supplemental Fig. 3). These results further confirm that mouse rIL-33 can promote systemic inflammation.

### Recombinant IL-33 treatment in mouse model of endometriosis stimulated proliferation and vascularization of the endometriotic lesions

**Figure 4:**
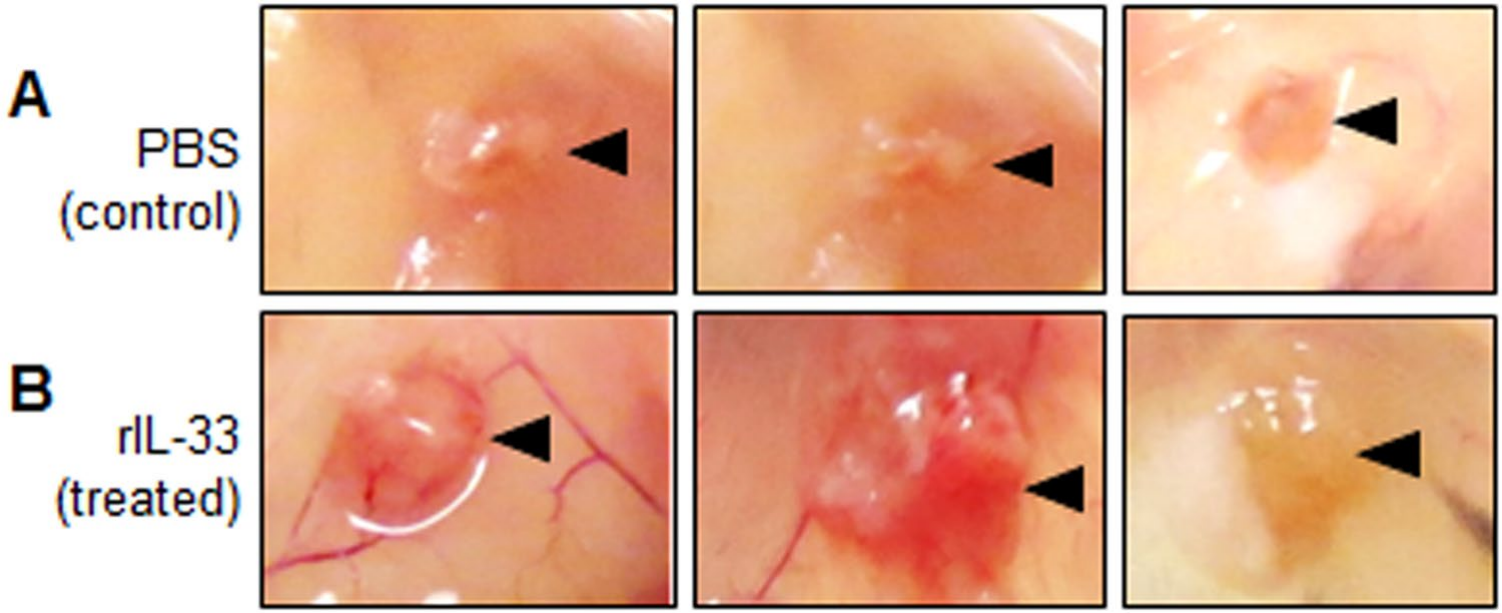
Endometriotic lesions harvested from PBS (control) and mouse rIL-33 (treated) mice. Endometriotic lesions harvested from mouse r-IL33 mice (n = 5) appear qualitatively larger with enhanced vasculature (**B**) compared to PBS treated (n = 5) mice (**A**). Black triangles indicate the endometriotic lesion.

**Figure 5:**
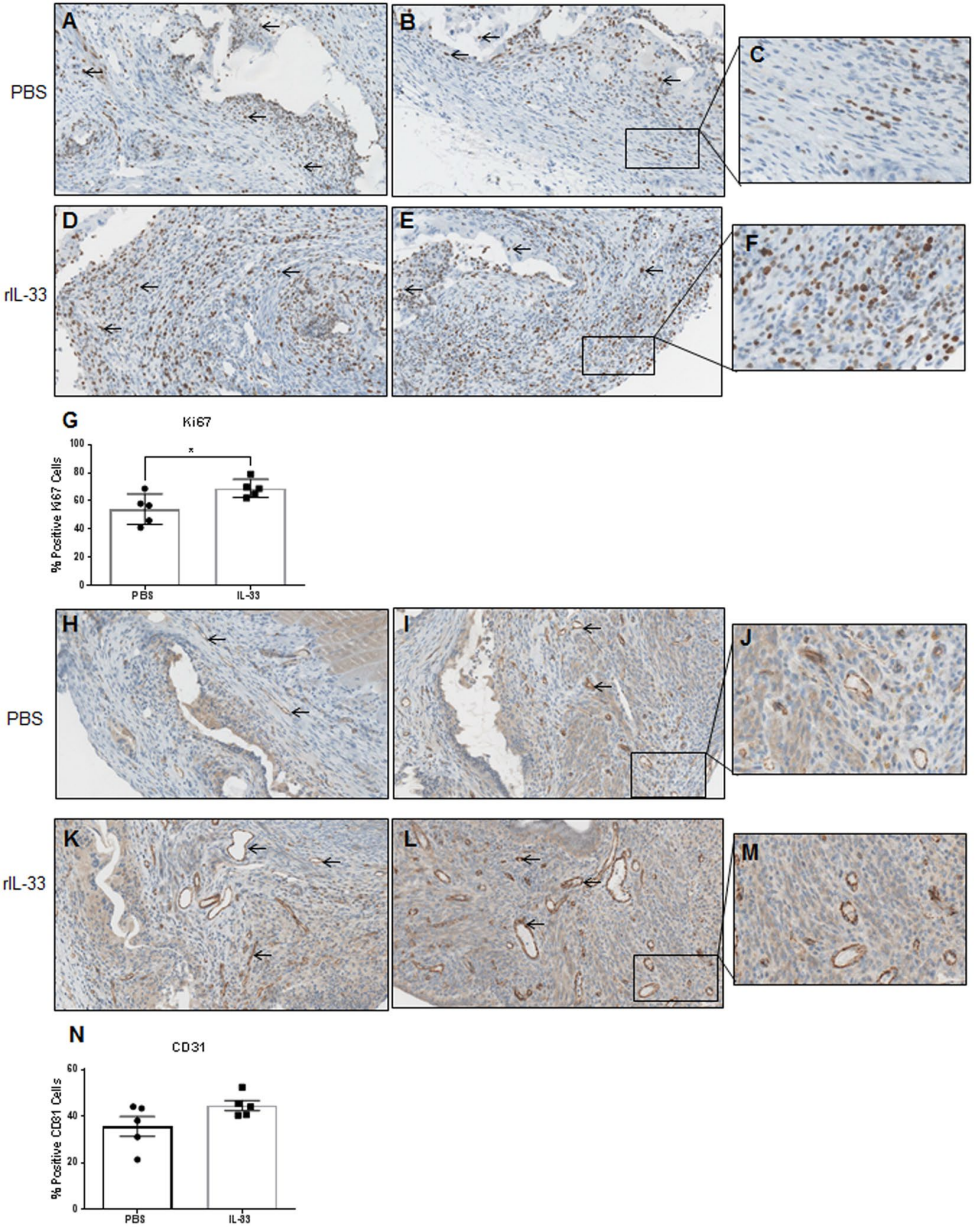
Immunohistochemistry with a proliferation marker, Ki67 (**A**–**F**) and vasculature marker CD31 (**H**–**M**) revealed intense immunostaining. Semi-quantitative analysis of Ki67 (**G**) showed significant increase in % positive Ki67 cells in the mouse rIL-33 treated mice compared to control. Semi-quantitative analysis of CD31 (**N**) immunostaining shows a higher trend in rIL33 treated mice compared to controls. Black arrows indicate positive staining. *p < 0.05 **p < 0.01 ***p < 0.0001.

As mentioned previously, hallmark features of endometriosis include the ability of the endometriotic lesions to grow and establish a blood supply. After treatment with mouse rIL-33, treated mice had qualitatively larger lesions and enhanced vascularization supplying the lesion compared to control (Fig. 4A,B). Endometriotic lesions obtained from both rIL-33 treated and control mice were evaluated using an immunohistochemistry for Ki67, the well-established cell proliferation marker, and CD31, a known marker of endothelial cells. Semi quantitative analysis of Ki67 showed that mice treated with rIL-33 had significantly increased staining compared to control mice (Fig. 5A–G). Additionally, Ki67 staining appeared to be abundant and widely dispersed in the lesions from rIL-33 treated mice; however, Ki67 staining in the lesions from PBS treatment mice appeared to be localized to certain areas. Quantitative analysis of CD31 indicated that mice treated with rIL-33 had a trend of increased CD31 compared to control; however, this was not statistically significant (p = 0.09, Fig. 5H–N).

## Discussion

IL-33 has emerged as a critical regulator of several chronic inflammatory diseases, autoimmune diseases and fibrotic disorders including asthma, rheumatoid arthritis, ulcerative colitis, lung fibrosis and cardiovascular disease^18,30,32–34^. However, the role of IL-33 in the progression of endometriosis is not well described. In this study, we set out to understand whether endometriotic lesions from patients produce IL-33 and express its receptor ST2 and to understand the mechanistic basis of their involvement in the pathophysiology of endometriosis using representative cell lines and a syngeneic mouse model.

From previous reports, we know that PF cells had significantly higher IL-33 mRNA^26^ suggesting that a variety of immune cells are producing IL-33 and potentially contributing to the high levels observed in PF^26^. To our knowledge, we show for the first time that endometriotic lesions from advanced stage patients had significantly higher levels of IL-33 compared to endometrial samples from healthy, fertile controls. This finding supports an earlier report showing higher concentrations of IL-33 in peritoneal and sera samples of subsets of patients with a deep infiltrating endometriosis phenotype^25^. Future studies are required to address the specific cell types in the endometriotic lesions as well as in the peritoneal cavity that are contributing to the elevated IL-33 levels in endometriosis patients. Additionally, we show that endometriotic lesions express ST2, suggesting that endometriotic lesions may respond to IL-33 signaling in an autocrine fashion. Previous reports found that endometriosis patients had significantly higher levels of sST2 in the PF compared to healthy controls^26^. Because we did not find significant differences between the levels of plasma sST2 in endometriosis patients compared to controls, we speculate that perhaps high levels of sST2 are localized to the peritoneal microenvironment in attempt to modulate the local, elevated levels of IL-33.

To gain further insights into IL-33 signaling in the progression of endometriosis, we studied the effect of human rIL-33 stimulation of endometrial and endometriotic epithelial and endothelial cell lines. Our results show that human rIL33 stimulated the production of pro-inflammatory and angiogenic cytokines and promoted tubulogenesis. This further confirms IL-33’s established function as a pro-inflammatory and angiogenic cytokine^29,31,35^. As mentioned previously, a pro-inflammatory response is critical for disease progression because the inflammation in the peritoneal cavity of endometriosis patients has been linked to chronic pain and infertility experienced by these patients^3,11^. Also, the ability for the endometriotic lesion to establish a blood supply is an important step in endometriosis development, as this allows the lesion to grow and develop after its initial adhesion^36^. Together, our data generated from *in vitro* studies suggests that IL-33 promotes inflammation and angiogenesis, which likely impacts the inflammatory *mileu* and disease progression in endometriosis patients.

Our patient data and *in vitro* data, along with previous reports, provide convincing evidence to suggest a potential role of IL-33 in endometriosis. Therefore, we used a syngeneic immunocompetent mouse model to understand the impact of an artificially elevated IL-33 microenvironment on the progression of endometriosis. Mice treated with mouse rIL-33 had significantly elevated levels of CXCL1, IL-6, GM-CSF, Eotaxin, IL-5, IL-7 and IL-33 in the plasma (Fig. 3) suggesting that IL-33 causes systemic inflammation in mice. In another study using a mouse model of allergic-induced inflammation, treatment with mouse rIL-33 polarized CD4 + T cells to produce IL-5 and facilitated the disease pathology by promoting a type 2 immune response and perpetuated airway inflammation^37^. Due to the upregulation of IL-5 in the plasma of the mice treated with mouse rIL-33, we can speculate that these mice are similarly exhibiting a type 2 immune response. Overall, mouse rIL-33 led to systemic upregulation of cytokines known to be involved in the progression of endometriosis^5,38^; however, we did not pin point which specific immune cell subsets or other cells were responding to the excess of rIL-33. When mice without endometriosis were injected with mouse rIL-33 or PBS, the plasma of mice treated with mouse rIL-33 had elevated levels of CXCL1, IL-6, GM-CSF, IL-7, IL-5 and Eotaxin, further suggesting that IL-33 likely perpetuates inflammation by interacting with immune cells rather than interacting with the endometriotic lesion itself. From previous study using other disease models, we can speculate that immune cells such as macrophages, T cells, innate lymphoid cells, and mast cells were likely involved in the progression of inflammation in response to rIL-33^19,32,39^.

One of the most important observations of our study was the localized effect of IL-33 on the proliferation and growth of the endometriotic lesion. Mice treated with mouse rIL-33 had larger and highly vascularized lesions compared to controls (Fig. 4). While the growth and size of the lesion is not always correlated with the symptoms of endometriosis, the goal of curing endometriosis would be to restrict growth and development of the lesion to the point where it ceased to exist. Contrary to our *in vivo* observation of increased proliferation of endometriotic lesions in response to IL-33 treatment, our *in vitro* data did not show any proliferative effects in the representative cell lines (endometrial epithelial, endothelial and endometriotic epithelial cell lines). This could simply be because of the complex composition of endometriotic lesions and perhaps a cross talk is present between cells within the lesion microenvironment causing increased proliferation *in vivo*. Additionally, the endometriotic lesions from mice treated with mouse rIL-33 had increased lymphocyte infiltration, suggesting a localized inflammatory response.

Interestingly, CXCL1, IL-6 and GM-CSF, were elevated in both the supernatant from endometriotic cell line (12Z) and in the plasma of mice treated with mouse rIL-33, which provides further validity to the effect of IL-33 in two different disease models. CXCL1, IL-6 and GM-CSF have been shown to be upregulated in the tissue and plasma of women with endometriosis compared to healthy fertile controls and suggests that IL-33 could be indirectly attracting neutrophils and macrophages to the microenvironment, through the upregulation of these cytokines and chemokines^5,40^. As mentioned earlier, with the ability to stimulate several innate and adaptive immune cells, IL-33 likely contributes to the progression of an inflammatory *milieu* through the activation of immune cells. However, these mechanisms need to be further studied in the context of endometriosis. The limitations of this study are common to studies that investigate endometriosis in general. First, the classification system of endometriosis is rudimentary due to the heterogeneity and complexity of the disease. Additionally, collecting endometriosis patient samples can be difficult due to the current diagnostic delay of 6–11 years and the invasive procedure required to obtain samples. Therefore, regardless of the prevalence of the disease, gathering well stratified samples from patients is an evolving challenge. Additionally, we used the syngeneic immunocompetent mouse model of endometriosis, which may not truly represent the human condition, but it provided us with the opportunity to gain mechanistic insights on the disease progression in an artificially created IL-33 dominant microenvironment. Additionally, we did not design experiments in the present study to account for the influence of intrinsic estrogen on IL-33 pathway. Estrogen has been shown to modulate levels of IL-33^41^. Despite these limitations, we have shown that human endometriotic lesions produce IL-33, express ST2 and that levels of IL-33 are significantly elevated in the endometriotic tissue of advanced staged patients compared to healthy, fertile controls. Additionally, we confirm previous reports that human rIL-33 initiates tube formation in endothelial cells and we show that human rIL-33 stimulates the production of angiogenic and pro-inflammatory cytokines. Finally, we show that in a syngeneic mouse model, elevated levels of mouse rIL-33 initiates local and systemic inflammation, stimulates the proliferation of the endometriotic lesion and induces angiogenesis. Overall, the present study provides evidence to suggest that IL-33 is likely one of the important players contributing towards inflammation observed in advanced staged endometriosis patients and the progression of the disease.

## Methods

### Ethics statement

Ethics was approved for this study by the Greenville Health System (South Carolina) and the University of North Carolina from Chapel Hill, USA. Human ectopic, eutopic endometrial tissue samples from endometriosis patients and control samples from fertile women were collected as per institutional approved protocols and guidelines. Written, informed consent was acquired before patient sample collection and storage. The Health Sciences Research Ethics Board, Queen’s University, Kingston approved ethics for this study. All methods were performed as per institutional approved guidelines.

### Analysis of sST2 in human plasma samples using an ELISA

Blood was collected in an EDTA-containing vacuum tube from 24 patients before undergoing surgery to ablate endometriotic lesions. Similarly, plasma was collected from 16 healthy, fertile controls free from the disease at the University of North Carolina School of Medicine. None of the healthy volunteers had signs or symptoms of endometriosis or infertility. Plasma samples were stored at −80 °C. Levels of ST2 were analyzed using an ST2 ELISA (R&D Systems; DST200). All reagents were prepared per the manufacturer’s protocol. In a 96 well microplate, 100uL of assay diluent was added and 50uL of the protein normalized sample or standard was added to each well and incubated for 2 hours at room temperature. Following aspiration and four washes, 200 uL of Human ST2 conjugate was added to each well and incubated for 2 hours at room temperature. Following washes, 200uL of substrate solution was added to each well and incubated for 30 mins at room temperature. After 30 mins, 50uL of stop solution was added and plates were read using a microplate reader set to 450 nm.

### Human endometrium (eutopic) and endometriosis (ectopic) tissue samples

Tissue samples were obtained from 20 endometriosis patients that underwent resection of endometriotic lesions due to endometriosis-associated infertility at Greenville Hospital after informed consent. All the patients enrolled in the study were free of any form of hormone therapy at least 3 months prior to surgery. Patients were grouped into disease stage (stage I–IV) as per American Society for Reproductive Medicine criteria. Patients were group as either early staged (stage I and II, n = 14) or advanced staged patients (Stage III and IV, n = 5). Endometrial samples (eutopic) were collected from endometriosis patients using pipelle biopsy. Similarly, endometrial biopsies were collected from 11 healthy, fertile controls free from the disease at the University of North Carolina School of Medicine. None of the healthy volunteers had signs or symptoms of endometriosis or infertility. Tissue samples were stored at −80 °C.

### Protein extraction from tissue samples and analysis of IL-33 and ST2 in tissue using a multiplex cytokine array and an ELISA

Total protein was extracted from eutopic and ectopic tissue (from endometriosis patients) and endometrial tissue samples (from healthy, fertile controls) Samples were placed in microcentrifuge tubes containing 500uL of tissue extraction reagent I (FNN0071; Thermo Fisher), 5 uL protease inhibitor (Sigma-Aldrich, St. Louis, MO, USA) and homogenized using a rotor-stator homogenizer on ice. The samples were centrifuged at 18000 RPM at 4 °C and the supernatants were collected. Protein concentrations were measured using a bicinchoninic acid (BCA) assay (Biorad, Mississauga, ON, CA). All samples were normalized. Levels of IL-33 were analyzed using a commercially available human multiplex cytokine assay from Eve Technologies, Calgary, AL, Canada. Levels of ST2 were analyzed using an ST2 ELISA (R&D Systems; DST200). All reagents were prepared per the manufacturer’s protocol, as explained above.

### Cell culture

Endometrial epithelial carcinoma cells (EECC, CRL-1671; American Type Culture Collection, Manassas, VA), human umbilical vein endothelial cells (HUVEC, 200–05 f; Cell Applications, San Diego, CA) and epithelial, endometriotic 12Zs (generously provided Professor Anna Starzinski-Powitz) were incubated at 37 °C and 5% CO_2_. EECCs were maintained in Dulbecco’s Modified Eagle’s Medium (D6429; Sigma Aldrich) supplemented with 10% Fetal Bovine Serum (FBS) and 1% penicillin and streptomycin (Sigma Aldrich). HUVECs were maintained in Endothelial Cell Growth Medium (211–500; Cell Application). 12Zs were maintained in DMEM/F-12 (11330; Thermo Fischer Scientific) supplemented with 10% FBS, 1% penicillin and streptomycin and 1x sodium pyruvate.

### RNA extraction and RT-PCR

Total RNA from the three cell lines (EECC, HUVEC and 12Z) was extracted using RNA extraction kit (Norgen Biotek, CA) using manufacturer’s instructions. Total RNA was reverse transcribed using First-strand cDNA synthesis kit (GE Healthcare Life Sciences, Canada) as per the manufacturer’s protocol. Primers were designed using Primer3 software (http://frodo.wi.mit.edu/primer3/) from human sequences available on NCBI’s Nucleotide. Real-time PCR was performed using plate-based LC-480 (Roche Diagnostics, Montreal, Canada)^42^. Relative quantification was performed using *ACTB* as a housekeeping control gene. Samples were run in triplicates. The run protocol for both genes used was: Denaturation: 95 °C, 15 min; Amplification: 45 cycles: 95 °C for 15 s, 55 °C for 30 s, 70 °C for 30 s; Melting Curve: 70–95 °C, at a rate of 0.1 °C per second. Data was analyzed using the ∆∆Ct method.

### Cell proliferation using IncuCyte Cell Confluence Assay

Cell proliferation was evaluated using the IncuCyte cell confluence proliferation assay methodology (IncuCyte ZOOM 2016A; Essen Biosciences Inc.), as conducted previously by our group^43^. EECC, HUVECs and 12Zs were harvested with 1x Trypsin-EDTA and 2.0 × 10^4^ cells/well were seeded onto a 24-well tissue-culture plate (Sarsted Newton), followed by human rIL-33 stimulation at 1, 10, 50, and 100 ng/ml (3625-IL-010; R&D Systems, Minneapolis, MN) in triplicates. PBS was used as a control. Cell confluence was measured over 48 hours.

### Cell culture supernatant cytokine analysis using multiplex array

EECCs, HUVECs and 12Zs were harvested with 1x Trypsin-EDTA and seeded at 10^5^ cells/well onto a six-well plate (Sarstedt Newton) and stimulated with 10, 50, and 100 ng/ml of human rIL-33 (3625-IL-010; R&D Systems, Minneapolis, MN) in triplicates. Concentrations of rIL-33 were selected based on the levels of IL-33 in the PF found previously^25^. PBS was used as control. The cells were incubated for 24 h at 37 °C with 5% CO_2._ The conditioned media was collected and analyzed using a commercially available human multiplex cytokine assay from Eve Technologies, Calgary, AL, Canada.

### Angiogenesis (tubulogenesis) Assay

The angiogenesis assay was performed as per manual instructions (R&D Systems; 3470–096-K). 50uL of Cultrex® RGF BME was aliquoted into each well of a 96 well plate and incubated at 37 °C for 30 minutes to allow the BME to gel. Then, HUVECs were harvested with 1x Trypsin-EDTA and seeded at 10^4^ cells/well and varying concentrations (10, 50, and 100 ng/mL) of human rIL-33 (3625-IL-010; R&D Systems, Minneapolis, MN) in triplicates. Cells were incubated for 6 hours in a CO_2_ incubator at 37 °C. Images were taken using IncuCyte ZOOM (2016A; Essen Biosciences Inc) and tube formation was evaluated and quantified using NIH ImageJ with the angiogenesis analyzer plugin^44^.

### Syngeneic mouse model of endometriosis

All animal experiments were performed under protocols approved by Queen’s University Institutional Animal Care Committee as per Canadian Council of Animal Care guidelines. Mature (8–10-week-old) C57Bl/6 mice (n = 15, Charles River, USA) were housed in cages of 3–4. After euthanasia, the uterine horns were harvested from donor mice (n = 5), and placed in a petri dish containing PBS. The endometrium was cut into 2 mm^3^ fragments using an epidermal puncher and kept on ice until they were surgically explanted into the recipient mice (n = 10). Under 4% isofluorane vaporizer anesthesia, small incisions were made in the abdomen of recipient mice and the donor endometrial fragments were adhered to the right side of the peritoneal cavity using bonding agent. Following surgery, the animals were rested for 14 days. Then, mice in the treatment group (n = 5) received intraperitoneal injections of mouse rIL-33 at 1ug/mouse in 100uL volume (Ebioscience; 14-8332-80) two times a week for two weeks. Mice from the control group (n = 5) received two intraperitoneal injections of PBS for two weeks. Blood was collected from all the experimental mice at day 7, day 18 and day 25, where day 0 indicates the date of surgery and day 15 indicates the onset of PBS or mouse rIL-33 treatment (Fig. 3A). Mice were sacrificed on day 25 and ectopic lesions, uterus, ovaries, spleen, liver, kidneys and heart were harvested, fixed using 4% paraformaldehyde, and stored in 70% ethanol prior to tissue processing.

To understand the effect of rIL-33 on mice without endometriosis, we injected 8–10 weeks old C57Bl/6 mice (n = 6, Charles River, USA) with mouse rIL-33. These healthy mice without endometriosis underwent biweekly intraperitoneal injections for one week with either PBS (n = 3) or mouse rIL-33 (1ug/mouse) in 100uL volume (n = 3). After one week, the mice were sacrificed and blood was collected by cardiac puncture. Plasma samples were analyzed using multi-plex cytokine assay from Eve technologies as explained below.

### Cytokine analysis in plasma samples from experimental mice using a multiplex array

100 uL of blood was collected from the submandibular vein at three time points (day 7, day 18 and day 25) in tubes coated with EDTA (K_2_EDTA; 365974) and immediately placed on ice. The blood was centrifuged for 15 mins at 3,000 rpm at 4 C, plasma collected and aliquoted for storage at −80C. Aliquots were diluted in PBS to a dilution factor of 2 and were analyzed using a mouse multiplex cytokine analysis from Eve Technologies, Calgary, AL, Canada as described previously^5^. Cytokines included in the multiplex panel were: Eotaxin, G-CSF, GM-CSF, IFN $\gamma$, IL-1a, IL-1b, IL-2, IL-3, IL-4, IL-5, IL-6, IL-7, IL-9, IL-10, IL-12 (p40), IL-12 (p70), IL-13, IL-15, IL-17A, IP-10, KC, LIF, LIX, MCP-1, M-CSF, MIG, MIP-1alpha, MIP-1beta, MIP-2, RANTES, TNFalpha, VEGF, IL-17E, IL-17F, IL-21, IL-22, IL-23, IL-27, IL-28B, IL-31, IL-33, and MIP-3a. Blood was collected from mice without endometriosis in tubes coated with EDTA (K_2_EDTA; 365974) and immediately placed on ice. Plasma was collected and stored at −80C. Aliquots were diluted in PBS to a dilution factor of 2 and were analyzed using the same mouse multiplex cytokine analysis from Eve Technologies, Calgary, AL, Canada.

### CD31 and Ki67 Immunohistochemistry

Paraformaldehyde fixed, paraffin embedded endometriotic lesions from mice were sectioned at 5 *μ*m thickness. After deparafinization in series of graded alcohols and citrosolve solution, sections were immunostained using a referral service with automated stainer in the Department of Pathology and Molecular Medicine at Queen’s University (BenchMark XT automated stainer from Ventana Medical System Inc., Tucson, AZ, USA). Following antigen retrieval with cell conditioning 1 for 60 min (Ventana Medical System Inc), sections were incubated with primary anti-CD31 (ab28364, Abcam 1:5000) and anti-Ki67 antibodies (ab16667, Abcam 1:1000). The sections were the stained with secondary antibodies for 60 min and Ultrablue DAB detection kit was used for color development (Ventana Medical System Inc). Finally, sections were counterstained with haematoxylin and bluing reagent for 4 min before adding the coverslips. For CD31 and Ki67 staining, differential cell counting analyses, image analysis software was conducted using NIH ImageJ as per previously published protocol from our group^45^. Briefly, 100 cells were manually counted from three areas from three different slides in each experimental group. All images had a total of 2014 × 1536 pixels each and were all from the same magnification (200 X).

### Statistical analysis

The matched eutopic and ectopic tissue from the same patient was analyzed using a paired t-test. Protein levels from patients compared to normal controls were analyzed using a one-way analysis of variance (ANOVA). The data collected the angiogenesis and the cell supernatant experiments was analyzed using a one-way analysis of variance (ANOVA). The data collected from the IncuCyte cell confluence proliferation assay and blood cytokine levels were analyzed using a two-way analysis of variance (ANOVA) with repeat measure. Differential cell counting experiments (CD31, Ki67) were compared using non-parametric student’s t-test to compare between treated and control groups. A p < 0.05 was considered as statistically significant. All statistical analysis was conducted and graphs were generated using Graphpad Prism 7.0 software (GraphPad Software Inc., California, USA).

## Electronic supplementary material

Supplemental Material
