# Supplementary material for: Interleukin-33 modulates inflammation in endometriosis
Source: Sci Rep. 2017 Dec 20;7:17903. doi: 10.1038/s41598-017-18224-x (PMC5738435; doi:10.1038/s41598-017-18224-x)

Interleukin-33 modulates inflammation in endometriosis

Jessica E Miller[1], Stephany P Monsanto[1], Soo Hyun Ahn[1], Kasra Khalaj[1], Asgi Fazleabas[2], Steven L Young[3], Bruce A Lessey[4], Madhuri Koti[1] and Chandrakant Tayade[1*]

Supplemental Material

Supplemental Figure 1. The effect of human recombinant IL-33 stimulation on the tube formation of HUVECs. All experiments were conducted in triplicates. **(A)** An image of HUVECs treated with PBS. **(B)** An image of HUVECs treated with 10ng/mL of human rIL-33. **(C)** The total branch length of the formed tubules. Upon stimulation with 10ng/mL of human rIL-33, the total branch length was significantly higher than the PBS treated cells. *p<0.05 **p<0.01 ***p<0.0001

Supplemental Figure 1

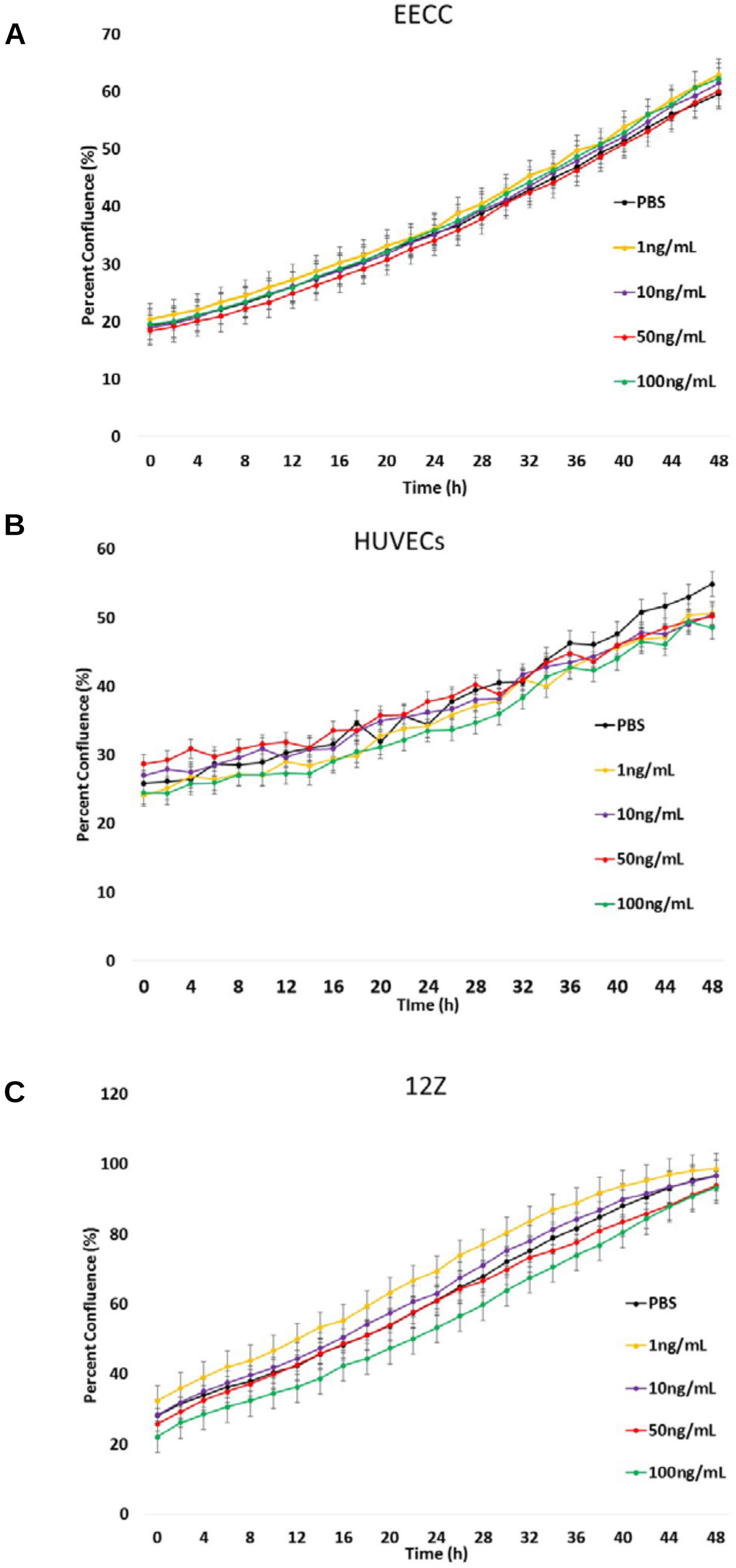

Supplemental Figure 2. The effect of human recombinant IL-33 stimulation on the proliferation of cell lines. All experiments were conducted in triplicates. **(A-C)** In response to varying concentrations of human rIL-33 (1, 10, 50 and 100ng/mL), the proliferation of endometrial epithelial carcinoma cells (EECC) and human umbilical vein endothelial cells (HUVEC) and endometriotic cells (12Z) did not change. *p<0.05 **p<0.01 ***p<0.0001

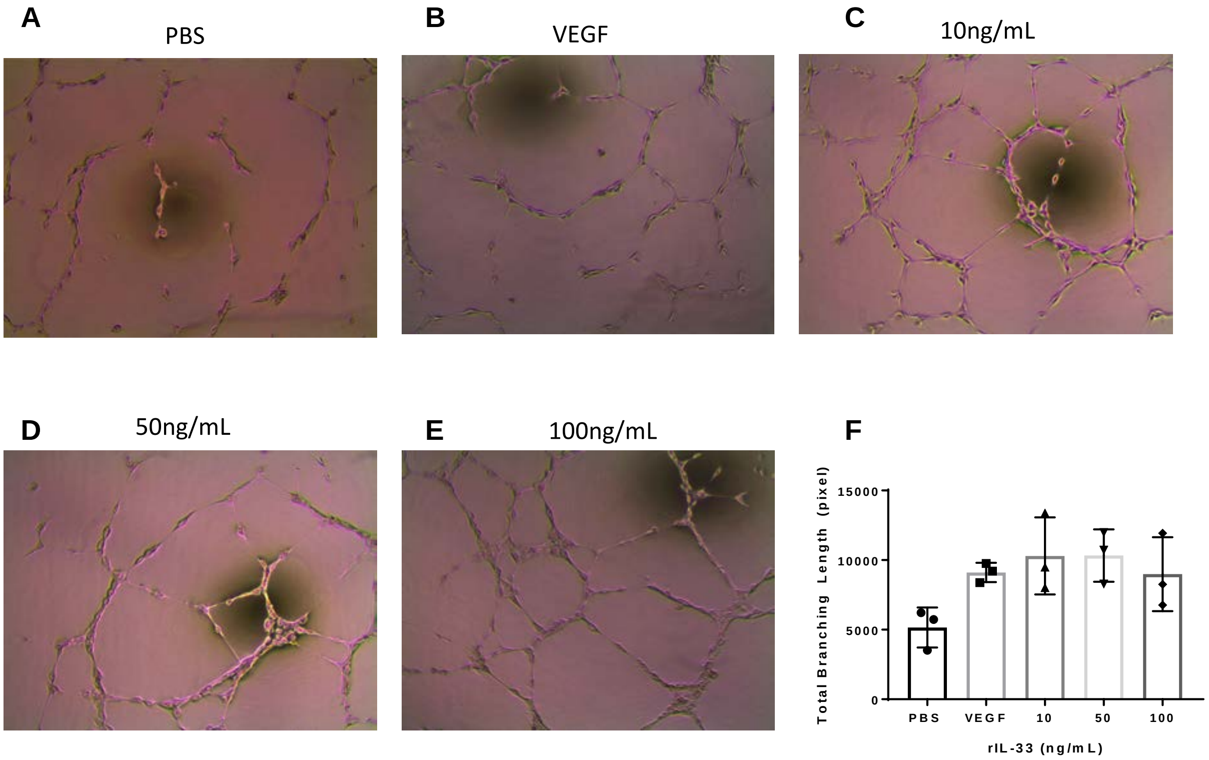

Supplemental Figure 3. Plasma cytokine profile in mice without endometriosis and treated with PBS (control) (n=3) or mouse rIL-33 (treated) (n=3). Plasma was analyzed using a mouse multiplex assay and non-significant cytokines are not shown. **(A-G)** Plasma cytokines revealed higher levels of Eotaxin, GM-CSF, IL-6, IL-7, IL-5, CXCL1, IL-17 and IL-33. *$p<0.05$

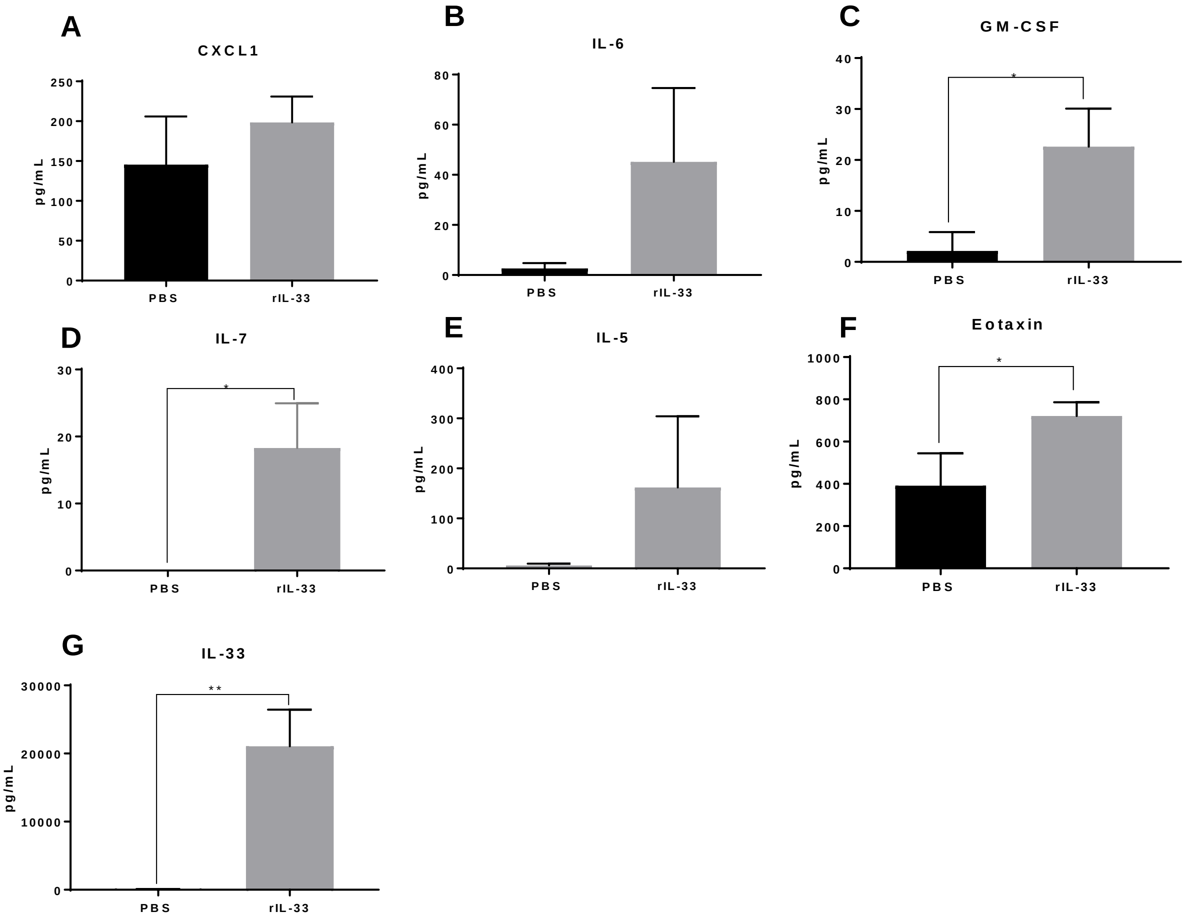

Supplement: Supplementary file 1 — Supplemental Material [file 41598_2017_18224_MOESM1_ESM.pdf]
